# Historical context, process, and development trends of pediatric thyroid cancer research: a bibliometric analysis

**DOI:** 10.3389/fonc.2024.1340872

**Published:** 2024-02-22

**Authors:** Chang Song, Jia-Yuan Luo, Yu-Yan Pang, Rong-Quan He, Xiao-Jiao Li, Gang Chen, Chun-Yan Zhao, Ning Qu, Yan-Mei Chen, Li Yang, Bi-Qi Li, Lin Shi

**Affiliations:** ^1^ Department of Pathology, Second Affiliated Hospital of Guangxi Medical University, Nanning, China; ^2^ Department of Pathology, First Affiliated Hospital of Guangxi Medical University, Nanning, China; ^3^ Department of Medical Oncology, First Affiliated Hospital of Guangxi Medical University, Nanning, China; ^4^ Department of Nuclear Medicine, First Affiliated Hospital of Guangxi Medical University, Nanning, China

**Keywords:** childhood thyroid cancer, bibliometrics, radiation exposure, genetic mutations, molecular targets

## Abstract

**Objective:**

At present, the structure of knowledge in the field of childhood thyroid cancer is not clear enough, and scholars lack a sufficient understanding of the developing trends in this field, which has led to a shortage of forward-looking outputs. The purpose of this research is to help scholars construct a complete knowledge framework and identify current challenges, opportunities, and development trends.

**Methods:**

We searched the literature in the Web of Science Core Collection database on August 7, 2023 and extracted key information from the top 100 most cited articles, such as the countries, institutions, authors, themes, and keywords. We used bibliometric tools such as bibliometrix, VOSviewer, and CiteSpace for a visualization analysis and Excel for statistical descriptions.

**Results:**

The top 100 most cited articles fluctuated over time, and the research was concentrated in European countries, the United States, and Japan, among which scientific research institutions and scholars from the United States made outstanding contributions. Keyword analysis revealed that research has shifted from simple treatment methods for pediatric thyroid cancer (total thyroidectomy) and inducing factors (the Chernobyl power station accident) to the clinical applications of genetic mutations (such as the *BRAF* and *RET* genes) and larger-scale genetic changes (mutation studies of the *DICER1* gene). The thematic strategy analysis showed an increasing trend towards the popularity of fusion oncogenes, while the popularity of research on traditional treatments and diagnostics has gradually declined.

**Conclusion:**

Extensive research has been conducted on the basic problems of pediatric thyroid cancer, and there has been significant outputs in the follow-up and cohort analysis of conventional diagnostic and treatment methods. However, these methods still have certain limitations. Therefore, scholars should focus on exploring fusion genes, the clinical applications of molecular targets, and novel treatment methods. This study provides a strong reference for scholars in this field.

## 1 Introduction

During the period from 2012 to 2016, thyroid cancer held a dominant position among pediatric endocrine cancers, with an incidence rate exceeding 6% of all childhood cancers. This data highlights a consistent upward trend in the incidence of pediatric thyroid cancer over the past four decades (1). Pediatric thyroid cancer patients have a poorer prognosis, higher rates of metastasis, and lower chances of survival. Research indicates that oncogenic gene fusions are more prevalent in pediatric thyroid carcinoma, with approximately 50–60% of pediatric patients exhibiting such fusions, compared to 15% in the adult population (2). Previous studies have shown that the causes of pediatric thyroid cancer are complex, including radiation exposure (3–6), iodine deficiency (7), and various non-exposure factors (8). Furthermore, there are quantitative and qualitative differences in gene mutations and growth factor expression patterns in pediatric thyroid cancer compared to the adult variant, as well as differences in symptoms and clinical manifestations, which pose challenges to diagnosis and treatment (9). Therefore, it is crucial to strengthen the research on and our understanding of pediatric thyroid cancer to improve patient survival and quality of life. Currently, with the increasing use of computed tomography (CT) imaging for pediatric patients and the growing psychological pressure they face (10, 11), new challenges are arising in the field of pediatric thyroid cancer.

Currently, a large number of original studies and clinical guidelines have accumulated in the field of pediatric thyroid cancer. The main research focuses on the fine-needle aspiration diagnosis, treatment strategies, and case analysis of pediatric thyroid cancer. However, due to the disorganized knowledge structure and lack of systematic reviews on this disease, there have been few prospective breakthroughs in recent years. At the same time, it is difficult to discern the historical evolution of and future trends in this field.

Bibliometric analysis can effectively address these issues. Bibliometrics holds significant importance in research fields. Through systematic analysis of factors such as document quantity, citations, and author relationships, bibliometrics can reveal trends in disciplinary development, research hotspots, and knowledge gaps, providing a comprehensive perspective for scientific research. This approach not only aids in assessing the quality and impact of research but also guides future research directions, fostering collaboration and communication between disciplines. In summary, bibliometrics serves as a crucial tool in the academic community, driving the development of scholarly research and optimizing resource allocation. Therefore, in this study, we performed a bibliometric analysis of the top 100 most cited articles on pediatric thyroid cancer to enhance researchers’ understanding of this field. We explored the studies’ basic information on research output, countries, institutions, authors, collaborations, thematic evolution, keyword coupling, and strategic analysis of topics, which will promote the development of research in this field and facilitate new discoveries in basic research (12).

## 2 Materials and methods

### 2.1 Data collection

We searched for papers in the field of pediatric thyroid cancer research through the Web of Science Core Collection database up to August 7, 2023. The search strategy was as follows: TS = [(“thyroid” OR “thyroid gland” OR “thyroidea” OR “glandula thyroidea” OR “TG”) and (“cancer” OR “tumor” OR “carcinoma” OR “neoplasm” OR “neoplasia” OR “malignancy” OR “malignant”) AND (“child” OR “children”)], with the results sorted in descending order of citation frequency. The research was independently screened by two investigators, who carefully read the titles, abstracts, and full texts of each paper to confirm their relevance to pediatric thyroid cancer research, and the disagreements were resolved by a third investigator. In the end, we identified the 100 most cited papers related to thyroid cancer in children. The literature was exported in full record formats, such as plain text files, BibTex, and Excel.

### 2.2 Data analysis

In this study, Bibliometrix (13), VOSviewer (14), and Citespace (15) were used for bibliometric analysis. The authors, institutions, countries, and publications were networked and visualized. Bibliometrix was used to build various publication trend charts and conduct a strategic theme analysis. VOSviewer was used for a collaborative network visualization analysis of the countries, institutions and authors. Citespace was used to analyze the countries, institutions, authors, topics, and keyword emergence for each paper. Descriptive statistics were calculated using Excel. These visualization analyses can help scholars determine the research structure, hot topics, and development trends in the field, providing guidance for future research.

## 3 Results

### 3.1 Basic information

**Figure 1:**
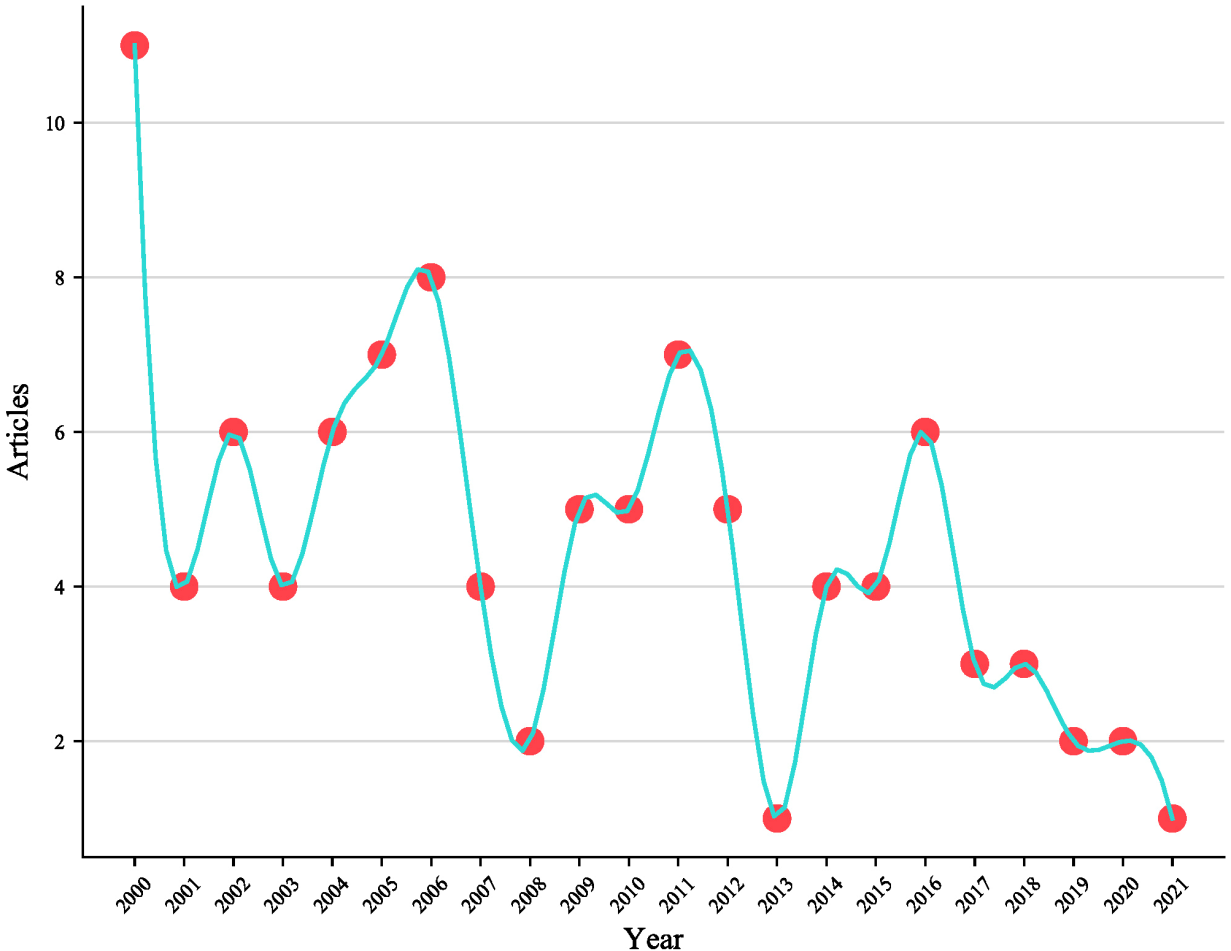
Trends in scientific output.

As shown in **Table 1**, the top 100 most cited articles include 74 original studies, 11 conference papers, and 15 review articles published from 2000 to 2021.The articles came from 50 different journals and involved 653 authors. The publication trend analysis showed that the largest number of articles were published in 2000, followed by a decline of -10.79% from 2000 to 2021. However, there were several significant fluctuations in 2006, 2011, and 2016 (**Figure 1**). In addition, the proportion of articles that involved international cooperations was as high as 38%.

**Table 1:** Main information of Childhood thyroid cancer papers included in this study.

Description	Results
MAIN INFORMATION ABOUT DATA	
Timespan	2000:2021
Sources (Journals, Books, etc)	50
Documents	100
Annual Growth Rate %	-10.79
Document Average Age	14.6
Average citations per doc	97.4
DOCUMENT CONTENTS	
Keywords Plus (ID)	365
Author’s Keywords (DE)	129
AUTHORS	
Authors	653
Authors of single-authored docs	3
AUTHORS COLLABORATION	
Single-authored docs	3
Co-Authors per Doc	8.66
International co-authorships %	38
DOCUMENT TYPES	
Article	74
Article; proceedings paper	11
Review	15

### 3.2 Research output at the national level

**Figure 2:**
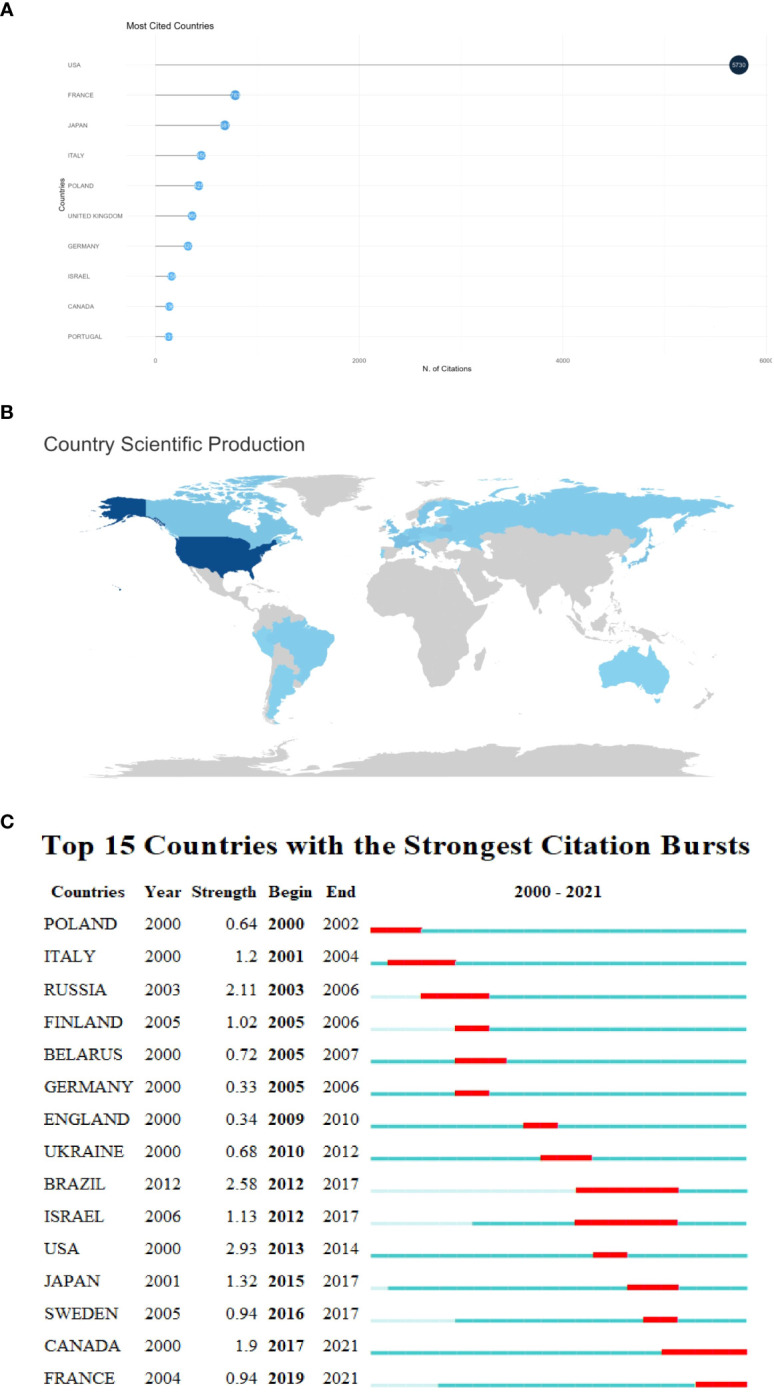
Country-level analysis: **(A)** Most cited countries. **(B)** Country research output map. **(C)** Country emergence.

In terms of citation frequency, the United States had the highest number of citations (n = 5730), far ahead of other countries, followed by France (n = 783) and Japan (n = 681) (**Figure 2A**). The scientific outputs are shown in **Figure 2B**. These research outputs were concentrated in European, North American countries and South American countries, of which the United States had the largest scientific output. Among Asian countries, only Japan devoted significant effort to research on childhood thyroid cancer. Additionally, some African countries and Australia were interested in this field. To explore the impacts of different countries over time, we conducted an emergent analysis of the top 15 most influential countries (**Figure 2C**). Around 2000, Poland, Italy, and Russia made significant contributions. Brazil and Israel had the largest influence between 2012 and 2017.

### 3.3 Scientific research output at the institutional level

**Figure 3:**
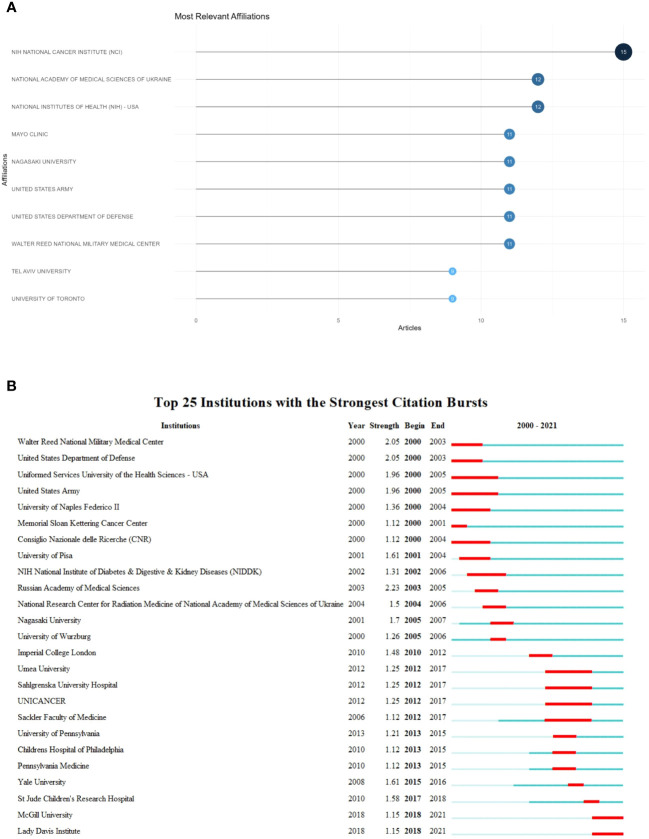
Institution-level analysis: **(A)** Top 10 most influential institutions. **(B)** Institutional emergence.

A visualization analysis of the top 10 research institutions, presented in **Figure 3**, showed that the National Cancer Institute (NCI) in the United States (n = 15) had the highest research output on pediatric thyroid cancer. Examining the top 25 of the 49 most influential institutions revealed that most of the research institutions were in the United States, such as the Walter Reed National Military Medical Center, United States Department of Defense, and Uniformed Services University of the Health Sciences, which contributed foundational research in the early development of this field. In these 21 years, institutions from the United States had a significant impact. In addition, Umea University, Sahlgrenska University Hospital, UNICANCER, and other scientific research institutions from European countries had long-lasting influence in this field.

### 3.4 Scientific research output at the author level

**Figure 4:**
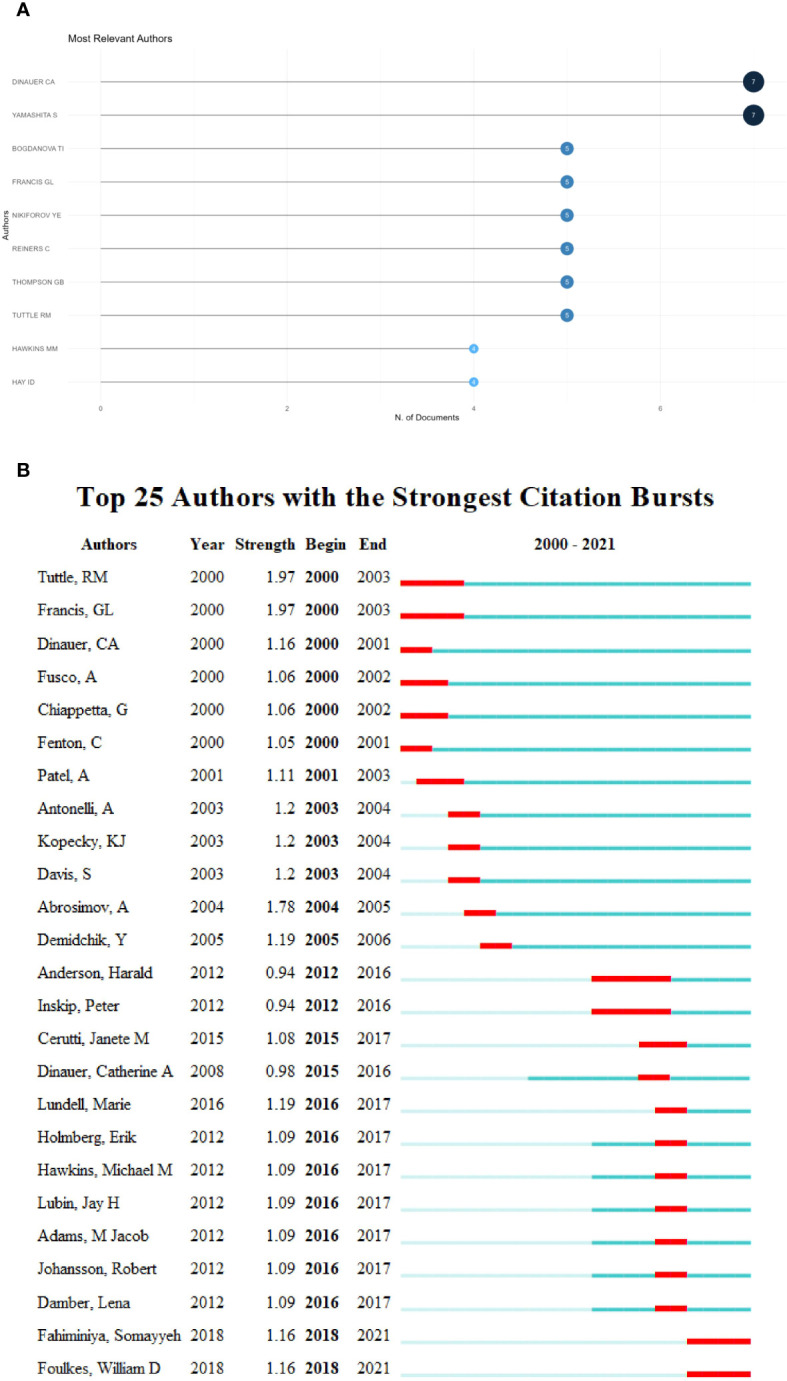
Author-level analysis: **(A)** Top 10 most relevant authors. **(B)** Author emergence.

A visualization analysis of the ten most relevant authors showed that Dinauer and Yamashita produced the most articles, with seven publications each. Bogdanova, Francis, Nikiforov, and other authors also made notable contributions (n = 5) (**Figure 4A**). Emergence analysis of the top 25 authors in **Figure 4B** revealed that Anderson and Inskip had the longest-lasting impacts. In recent years, Fahiminiya and Foulkes have made significant contributions.

### 3.5 Cooperation

**Figure 5:**
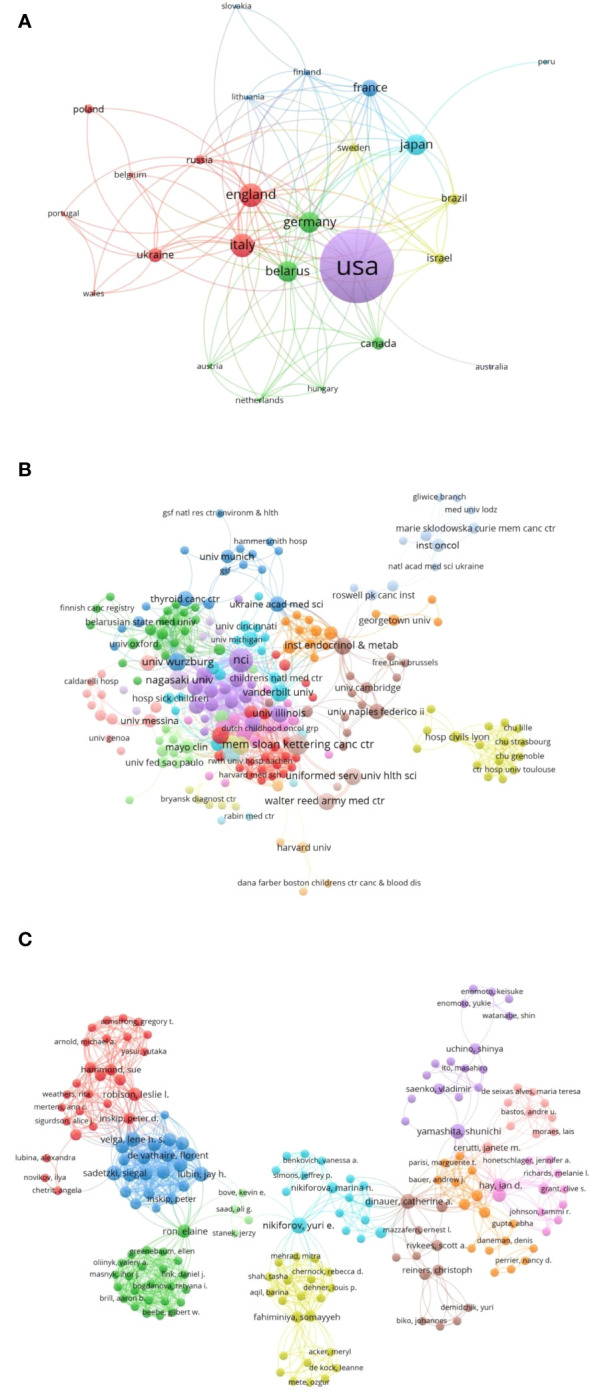
Cooperative networks: **(A)** Country cooperation. **(B)** Institutional cooperation. **(C)** Author cooperation.

We built a network of cooperation at the countries (**Figure 5A**), institutions (**Figure 5B**), and author (**Figure 5C**) levels, with connections between nodes representing cooperative relationships and node sizes representing the corresponding frequency of occurrences. We found that the cooperation between countries was relatively close, with strong collaboration among countries such as the United States, Germany, England, Italy, Japan, and Belarus. Institutions worked even more closely together than countries did, and at the center of the cooperative network was the NCI. The cooperation among authors was distributed in clusters, with a total of 10 clusters.

### 3.6 Theme and keyword analysis

#### 3.6.1 Evolution of themes and keywords

**Figure 6:**
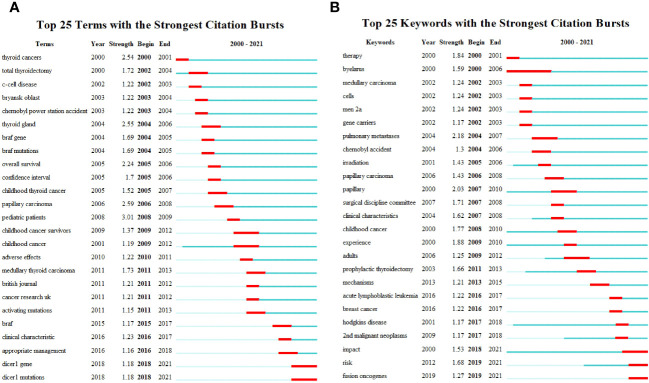
Emergence analysis: **(A)** Theme emergence. **(B)** Keyword emergence.

We further analyzed the evolution of historical hot topics and keywords as well as the transformation of knowledge. A comprehensive examination of the 100 studies and analysis of sudden changes in themes showed that the research touched on total thyroidectomy, the Chernobyl power station accident, some unique molecular pathological changes (the *BRAF* and *RET* genes), and clinical characteristics of the disease (overall survival and confidence interval). Later, the focus shifted to the clinical applications of gene mutations and a broader range of characteristic gene mutations, such as the *DICER1* gene and activating mutations (**Figure 6A**). Based on the theme evolution, we further explored the keyword evolution (**Figure 6B**), which showed a trend that was similar to that of the topic evolution. The keyword “mechanisms,” proposed in recent years, deserves special attention.

#### 3.6.2 Keyword coupling

**Figure 7:**
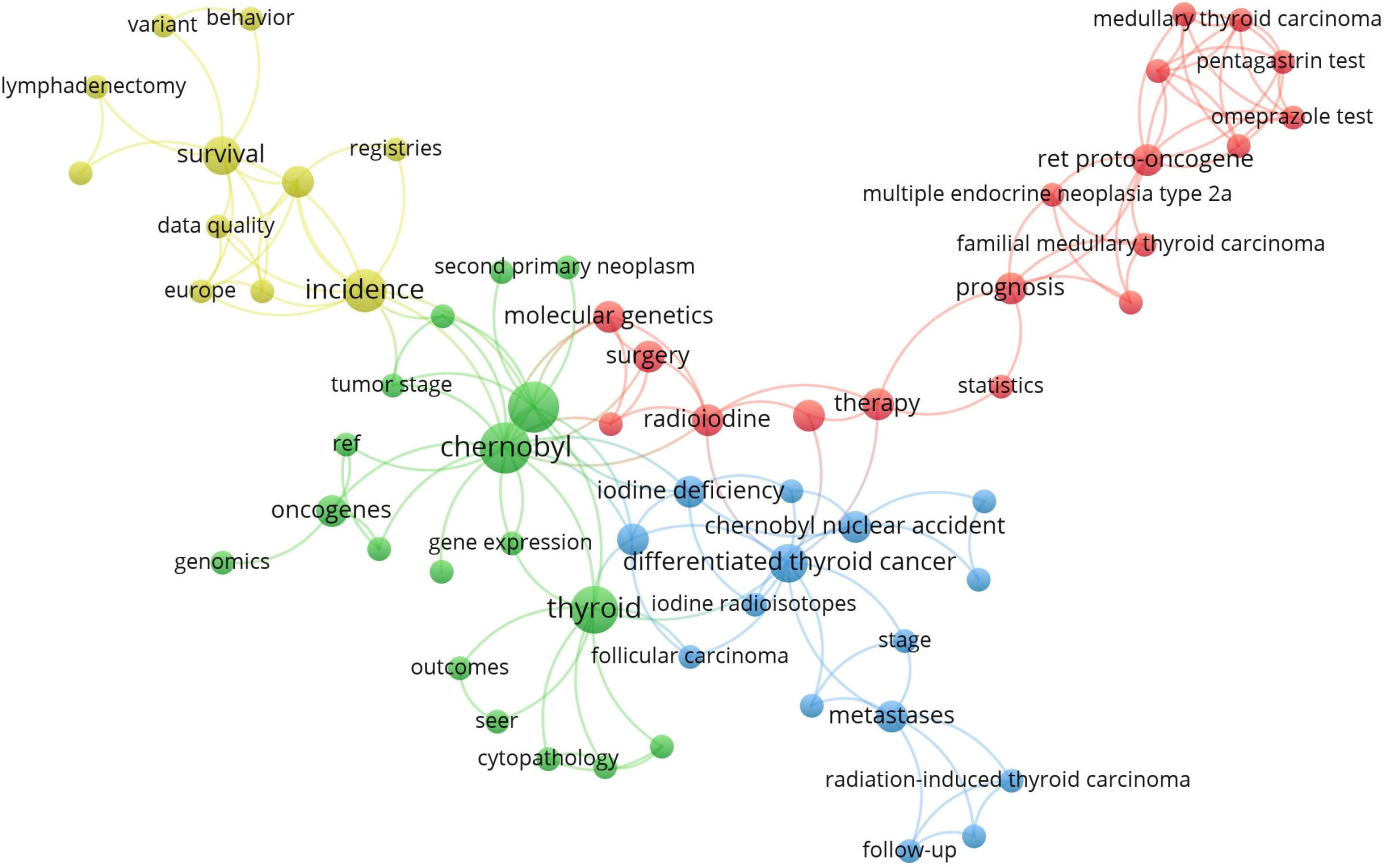
Keyword coupling network.

To accurately reproduce the knowledge composition and research framework of childhood thyroid cancer, 62 high-frequency keywords were extracted from 100 articles to construct a coupling network (**Figure 7**). The coupled networks were divided into four clusters: a green cluster for causes of thyroid cancer in children (such as the Chernobyl incident, oncogenes, and gene expression); a red cluster for the diagnosis and treatment of thyroid cancer in children (such as therapy, surgery, radioiodine, and the omeprazole test); a blue cluster for different types of childhood thyroid cancer (such as differentiated thyroid cancer, radiation-induced thyroid carcinoma, and follicular carcinoma); and a yellow cluster for clinical survival analysis and related experiments (on survival, data quality, registries, etc.).

#### 3.6.3 Thematic strategic analysis

**Figure 8:**
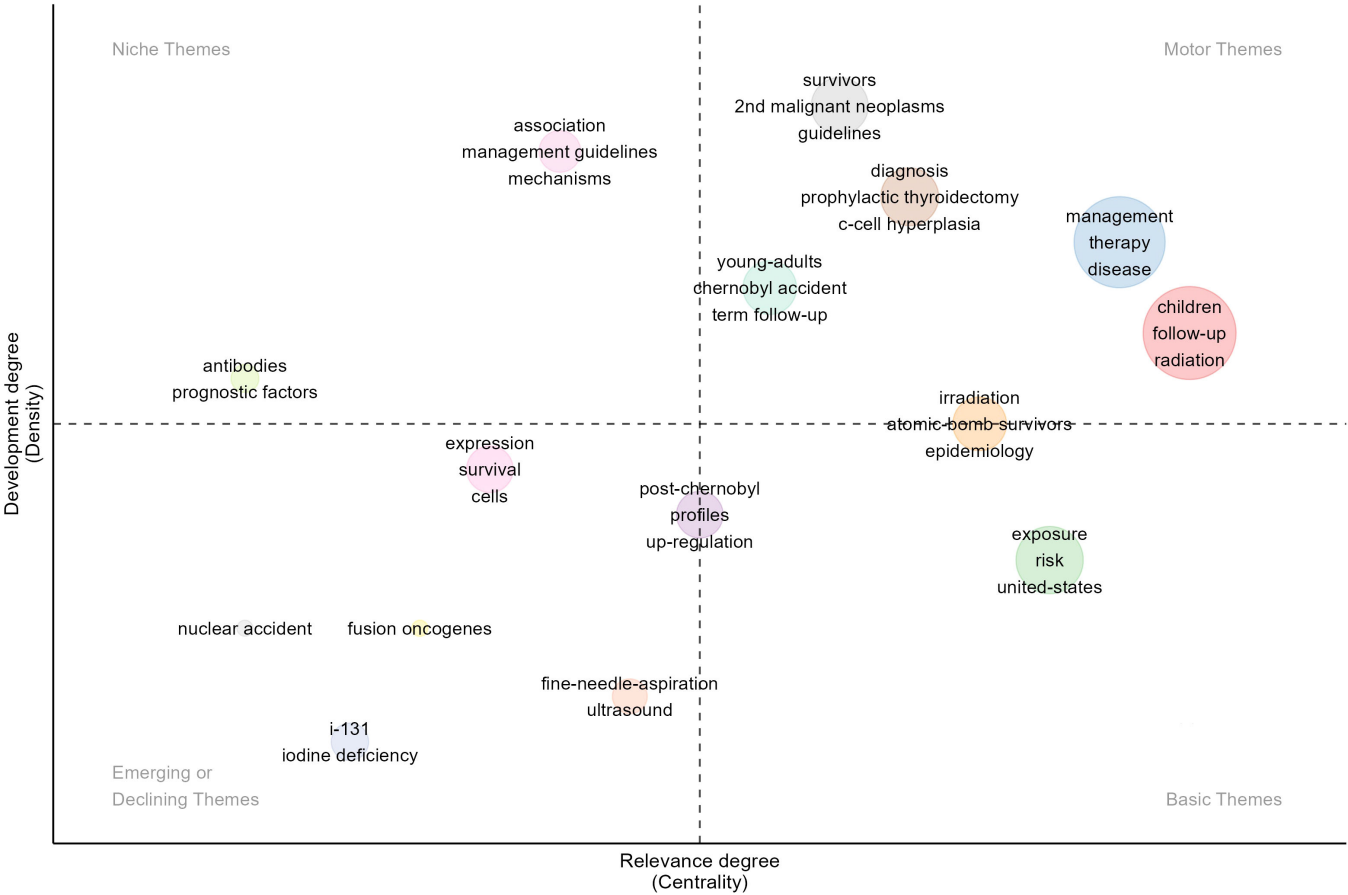
Thematic strategic analysis.

Finally, to help scholars accurately determine the direction of the evolution of childhood thyroid cancer and the development potential of different themes, we conducted a thematic strategic coordination analysis (**Figure 8**). The results showed that there were five clusters located in the first quadrant; with high centrality and density, these were the hotspots and core themes. These themes were summarized as follow-up after radiotherapy, treatment management, diagnosis and prevention, and secondary primary tumors. The second quadrant represented isolated themes with strong internal but weak external connections, including “antibodies,” “prognosis factors,” and “mechanisms.” The themes in the third quadrant had low centrality and density, indicating emerging or declining topics, such as “nuclear accident,” “fusion oncogenes,” “i-131,” “iodine deficiency,” “fine-needle-aspiration,” and “ultrasound”. The basic themes in the fourth quadrant had high centripetal degrees, including “exposure” and “risk”. Additionally, we observed that there were two class clusters at the intersection of two quadrants. “Atomic-bomb survivors” and “irradiation” were at the intersection of the first and fourth quadrants, indicating increasing research interest. “Post-Chernobyl,” “profiles,” and “up-regulation” were at the intersection of the third and fourth quadrants.

## 4 Discussion

**Figure 9:**
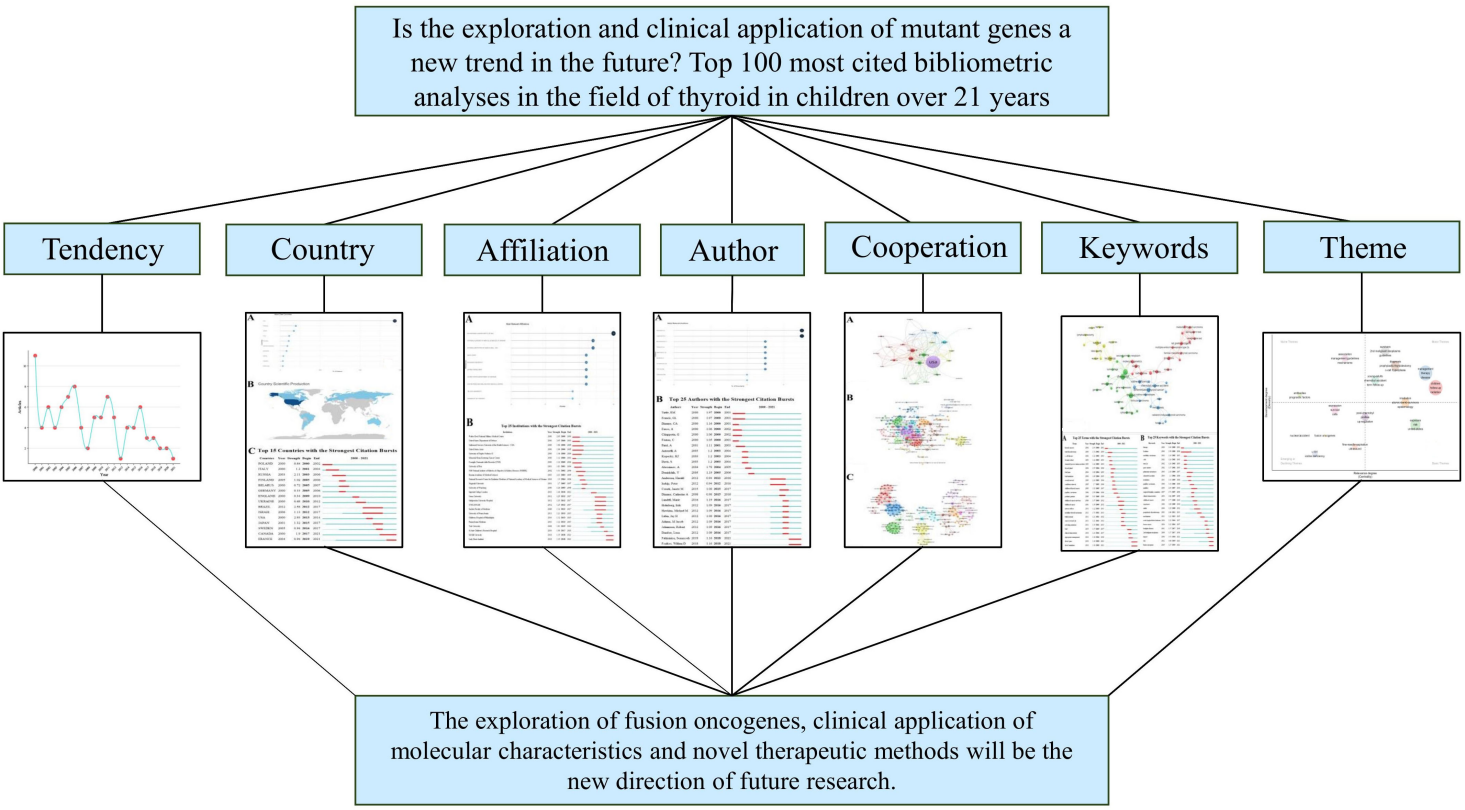
Schematic diagram of the research.

Bibliometrics is the systematic study of academic publishing and uses statistics to describe publishing trends and highlight relationships between published works (10). We used bibliometric research to visualize the overall publications and the countries, institutions, authors and partnerships, changes in thematic trends, and potential future directions of the top 100 most cited articles in the field of childhood thyroid cancer (**Figure 9**). More importantly, we aimed to provide a comprehensive reference for relevant clinicians and researchers to help them identify the most active scientific frontiers.

### 4.1 Research highlights

This study conducted a thorough and comprehensive analysis of the field of pediatric thyroid cancer. From the perspective of basic research information, the study included the top 100 most-cited articles from 2000 to 2021, covering various types of literature, journals, and research authors, revealing the developmental trajectory of this field. The publication trend showed a decline after reaching its peak in 2000, but the fluctuations indicated the ongoing vitality of the field. Moreover, the high percentage of international collaboration reflects a global interest in pediatric thyroid cancer research. The United States emerged as a leader in pediatric thyroid cancer research, with significant research outputs also observed in other countries such as France and Japan. The National Cancer Institute dominates this field, while some research institutions in European countries have had a lasting impact. Dinauer and Yamashita made remarkable contributions, and the sustained influence of Anderson Harald and Inskip Peter signifies their long-term contributions to the field. Collaboration among countries, institutions, and authors is tight, forming a complex collaborative network. Theme and keyword analysis revealed the evolution of pediatric thyroid cancer research from treatment methods and molecular pathological changes to broader characteristic genetic mutations. The introduction of the keyword “mechanisms” has garnered widespread attention. Finally, the strategic analysis of themes provides comprehensive insights into the field of pediatric thyroid cancer, offering crucial references for future research directions and strategies. In the following sections, we provide a detailed description and discussion of the aforementioned research results.

### 4.2 Temporal distribution of research output

From the perspective of publication trends and basic research conditions, the publication trend of childhood thyroid cancer research has exhibited significant fluctuations, especially in 2000, 2006, 2011, and 2016. Based on the strong association between radiation exposure and the risk of thyroid cancer in children (16), we speculate that these fluctuations are related to the 1999 nuclear leak in Japan, the 2005 leak of large amounts of radioactive liquid from the largest nuclear power plant in the United Kingdom, the 2011 Fukushima nuclear leak in Japan, and the radioactive iodine leak from a Norwegian nuclear reactor in 2016.

### 4.3 Analysis at the national, institutional, and author levels

European and American institutions and scientists have made especially important contributions to this research. The NCI has had the largest influence of any research institution and occupies a central position in the research co-authorship network. This is largely due to the agency producing the earliest systematic study of thyroid cancer risk in children and exposure to a dose of I^131^ (17–22). In addition, it may be related to the breakthrough achievements of the NCI in the detection of genetic mutations in thyroid cancer, such as the discovery that *RET* oncogene mutations contribute to the use of prophylactic thyroidectomy in children (23). Anderson and Inskip have the longest-lasting research influence in the field. The two have devoted years of research to the risk of thyroid cancer after radiotherapy in children and to cohort analyses, finding that the risk of thyroid cancer after radiotherapy for childhood cancer is significantly increased (6, 24–30). European countries and Japan have also made great contributions to this research field. This is due to the large number of children exposed to radiation and the large increase in radioactive iodine intake after the Chernobyl and Fukushima accidents (18, 31, 32). The cooperation network among authors at the national and institutional levels shows that the research is networked and cooperative, which suggests that scholars interested in this field should strengthen cooperative research. At the same time, researchers can also reference this research to find potential high-quality partners.

### 4.4 Analysis of keywords and topic trends

The historical context, research process, and development trends of childhood thyroid cancer are the main focus of this study. Based on the evolution, emergence, and thematic strategy analysis of theme keywords, it can be observed that the research ranges from simple treatment methods and predisposing factors of pediatric thyroid cancer to its clinical characteristics, and then further in-depth to gene mutations, molecular mechanisms, and biomarker detection. Four years after the Chernobyl nuclear accident, a dramatic increase in childhood thyroid cancer was observed in Belarus and Ukraine, and its causes; screening and treatment methods; prognosis; and clinical features need to be identified (18, 33–36). Initially, age, radiation exposure, disease risk, disease severity, and probability of recurrence were strongly correlated, suggesting that children are at higher risk of radiation-related thyroid cancer than adults (37–39). Early studies have reported routine examinations of thyroid nodules, such as fine needle aspiration and neck ultrasound (40–42). Treatment has mainly concentrated on total thyroidectomy, radioactive iodine therapy, and follow-up or related cohort analyses  throughout the study phase. Unfortunately, both surgical methods seem to have certain drawbacks. Total thyroidectomy is considered the best treatment option to some extent (43), but postoperative effects may persist, leading to complications such as hypocalcemia, hematoma, recurrent laryngeal nerve injury, and hypoparathyroidism (44, 45). In differentiated thyroid cancer in childhood, radioactive iodine therapy provides specific survival benefits (46), but there is an increased risk of a second primary malignancy and radiation fibrosis after treatment (47). In earlier years, scholars noted characteristic genetic mutations related to childhood thyroid cancer. Radiation-specific thyroid cancer in children has been associated with ret/PTC rearrangements occurring more frequently and generates new rearrangements (PTC8) in radiation-induced childhood thyroid cancer, and it is specific to this age group (48). In addition, numerous studies have discovered that *BRAF* mutations, which are common in adult thyroid cancer, are not the predominant molecular event in pediatric thyroid cancer (49–51). Unfortunately, the research at that time was limited to the mutation expression of *BRAF*, but did not explore its clinical application or its relationship with survival prognosis. Subsequently, under the influence of nuclear leakage incidents around the world, childhood thyroid cancer has gradually attracted the attention of more scholars globally, and the research has moved beyond characterization towards breakthroughs in treatment, diagnosis, and genetic mechanisms (52–54). In terms of treatment, for example, vandetanib targeted therapy has emerged as a highly active new treatment for children and adolescents with locally advanced or metastatic medullary thyroid cancer who tolerate it well (55). More and more scholars are focusing on the study of the molecular mechanism and gene expression of thyroid cancer in children. In radiation-induced childhood thyroid cancer, many regions of copy number alteration have been detected in specific genes or chromosomes in the genome, which may serve as potential markers (56). In addition, the increase in chromosomal band *7q11* and specific overexpression of genes such as *CLIP2* after exposure to low doses of radiation may be specific molecular markers of thyroid cancer in children (57). Therefore, regarding the theme of the third quadrant of the thematic strategy map, it can be confirmed that with the continuous development of molecular targets, traditional diagnostic treatments, such as fine-needle-aspiration, ultrasound, and i-131 are diminishing, and fusion oncogenes are the subject of increasing research. The clinical pathological differences observed between children and adults may be attributed to different genetic alterations, of which fusion oncogenes are the main type, and fusion gene expression and aggressiveness are closely related (58, 59).

However, from the above research progression, it can be seen that effective clinical treatments for childhood thyroid cancer need to be explored to reduce side effects. At the same time, the potential value of molecular targets in the diagnosis of thyroid cancer in children is worth further exploiting.

### 4.5 Limitations of the study

Inevitably, there are certain limitations to this study. It only includes literature in English, and there may thus be a limited representation of high-quality literature in other languages. In addition, high-quality prospective studies published in recent years were not considered because of their recent appearance and low citation frequency. Importantly, it doesn’t imply lower quality or importance of articles from 2022 and 2023 compared to those from 2000 to 2021. It only reflects that, at data cutoff, recent articles hadn’t accumulated enough citations. In the future, we will conduct a larger bibliometric analysis to solve the above problems.

## 5 Conclusion

A bibliometric analysis of the top 100 most cited papers over the past 21 years found significant correlations between the research hotspots and outputs and the country or time of radiation leaks. Many nuclear leak incidents in the world have led to large-scale outbreaks of childhood thyroid cancer, but its unique molecular clinical characteristics have not been widely considered in diagnosis, treatment, and prognosis. Conventional treatment methods still have certain limitations. With the increasing use of CT imaging in pediatric patients and the growing psychological stress faced by children, pediatric thyroid dysfunction and thyroid cancer in children may face greater challenges. This study aims to help scholars worldwide better understand the developmental trends in pediatric thyroid cancer research, focusing on exploring fusion genes, clinical applications of molecular features, and novel treatment methods, in order to address this global issue as soon as possible.

## Author contributions

CS: Formal Analysis, Visualization, Writing – original draft. J-YL: Methodology, Resources, Software, Supervision, Writing – review & editing. Y-YP: Funding acquisition, Methodology, Resources, Software, Supervision, Writing – review & editing. R-QH: Methodology, Supervision, Writing – review & editing. X-JL: Methodology, Supervision, Writing – review & editing. GC: Conceptualization, Data curation, Funding acquisition, Project administration, Validation, Writing – review & editing. C-YZ: Formal Analysis, Investigation, Visualization, Writing – original draft. NQ: Formal Analysis, Investigation, Visualization, Writing – original draft. Y-MC: Formal Analysis, Investigation, Visualization, Writing – original draft. LY: Conceptualization, Data curation, Formal Analysis, Project administration, Validation, Writing – review & editing. B-QL: Conceptualization, Data curation, Formal Analysis, Project administration, Writing – review & editing. LS: Formal Analysis, Project administration, Validation, Writing – review & editing.
